# *In Vitro* Culture-Induced Pluripotency of Human Spermatogonial Stem Cells

**DOI:** 10.1155/2013/143028

**Published:** 2012-12-24

**Authors:** Jung Jin Lim, Hyung Joon Kim, Kye-Seong Kim, Jae Yup Hong, Dong Ryul Lee

**Affiliations:** ^1^Fertility Center of CHA Gangnam Medical Center, College of Medicine, CHA University, 606-5 Yeoksam-dong, Gangnam-gu, Seoul 135-081, Republic of Korea; ^2^Department of Anatomy and Cell Biology, College of Medicine, Hanyang University, Seoul 133-791, Republic of Korea; ^3^Department of Urology, CHA Bundang Medical Center, CHA University, Seongnam 463-712, Republic of Korea; ^4^Department of Biomedical Science, College of Life Science, CHA University, Seoul 135-081, Republic of Korea

## Abstract

Unipotent spermatogonial stem cells (SSCs) can be transformed into ESC-like cells that exhibit pluripotency *in vitro*. However, except for mouse models, their characterization and their origins have remained controversies in other models including humans. This controversy has arisen primarily from the lack of the direct induction of ESC-like cells from well-characterized SSCs. Thus, the aim of the present study was to find and characterize pluripotent human SSCs in *in vitro* cultures of characterized SSCs. Human testicular tissues were dissociated and plated onto gelatin/laminin-coated dishes to isolate SSCs. In the presence of growth factors SSCs formed multicellular clumps after 2–4 weeks of culture. At passages 1 and 5, the clumps were dissociated and were then analyzed using markers of pluripotent cells. The number of SSEA-4-positive cells was extremely low but increased gradually up to ~ 10% in the SSC clumps during culture. Most of the SSEA-4-negative cells expressed markers for SSCs, and some cells coexpressed markers of both pluripotent and germ cells. The pluripotent cells formed embryoid bodies and teratomas that contained derivatives of the three germ layers in SCID mice. These results suggest that the pluripotent cells present within the clumps were derived directly from SSCs during *in vitro* culture.

## 1. Introduction

Embryonic germ cells (EGCs), which can be derived from fetal unipotent primordial germ cells (PGCs), are pluripotent and have expression patterns of cell surface and gene markers similar to those of embryonic stem cells (ESCs). These markers include alkaline phosphatase, OCT-4, SSEA-4, NANOG, TRA-1-60, and REX-1. Other important characteristics, such as multicellular colony formation, maintaining normal and stable karyotypes, the ability to proliferate continuously, and the ability to differentiate into all three embryonic germ layers, can be acquired during *in vitro* induction [1]. Although it has been suggested that PGCs are typically unipotent and are able to produce only germ cells [2], several studies have shown that a small number of PGCs express OCT4 and NANOG during various stages of prenatal development. These results provide evidence that there exists a population of multipotent PGC. In contrast to the induction of pluripotent stem cells (iPSCs), this type of induction was a solely culture-induced procedure and did not rely on the introduction of exogenous transcription factors.

Spermatogonial stem cells (SSCs) are derived from PGCs during the neonatal period and can self-renew and produce large numbers of differentiating germ cells that become spermatozoa throughout adult life. Recently, some groups reported that SSCs obtained from neonatal and adult mouse testes can be induced to form multipotent SSCs (mSSCs) or multipotent germline stem cells (mGSCs) during *in vitro* culture, and these cells may have a pluripotency similar to that of ESCs [3, 4]. In mice, mSSCs (mGSCs) are phenotypically similar to ESC/EG cells except with respect to their genomic imprinting pattern. These stem cells can differentiate into various types of somatic cells *in vitro* and can produce teratomas *in vivo *[3]. These multipotent cells were isolated and established from adult mouse testes using genetic selection, and the rate of establishment of cell lines was about 27% [4]. Additionally, another group established a similar type of multipotent cells derived from GPR125^+^ spermatogonial progenitor cells, and derivatives of the three germ layers (contractile cardiac tissues *in vitro* and formed functional blood vessels *in vivo*) have been generated [5]. These results suggest that stem cells in the germline lineage may retain the ability to generate multipotent cells [4].

Conrad et al. reported that human adult germ-line stem cells (haGSCs) from testicular tissue can be induced to form ESC-like cells that display multipotency *in vitro *[6]. Several researchers have also reported the establishment of human mSSC lines with different morphologies in previous papers [7–9]. Those studies were performed with various culture systems, and the findings for human adult testicular tissue remain questionable [10–12]. In 2010, Ko et al. reported that clusters of cells from human testicular fibroblasts (hTFCs) can be easily established from human testicular cultures. Those cells that were not pluripotent were found to be morphologically similar to haGSCs [13]. It was believed that the controversy regarding the characterization of human mSSCs was primarily due to a lack of a protocol for direct induction from well-characterized SSCs. Recently, our group reported that highly pure human SSCs were isolated using a gelatin/laminin-coated dish. These cells proliferated under exogenous feeder-free culture conditions, and then their functions were characterized [14]. The aim of the present study was to identify and isolate human pluripotent SSCs derived from the long-term *in vitro *culture of well-characterized human SSCs.

## 2. Materials and Methods

### 2.1. Patient Samples

Testicular tissues were donated from obstructive azoospermic (OA) patients subjected to multiple testicular sperm extraction (TESE)-intracytoplasmic sperm injection (ICSI) treatment. When sperm were found in the dissected samples, the testicular material remaining after clinical requirements was donated for this study after obtaining the patient's consent. This study was approved by the Institutional Review Board of the CHA Gangnam Medical Center, Seoul, Korea.

### 2.2. Isolation and *In Vitro* Culture of SSCs

The isolation and culturing of human SSCs was performed as described in our previous report [14]. Briefly, the testicular tissues of 18 OA patients were placed in 10 mL of enzyme solution A containing 0.5 mg/mL type I collagenase (Sigma-Aldrich, St. Louis, MO), 10 *μ*g/mL DNase I (Sigma-Aldrich), 1 *μ*g/mL soybean trypsin inhibitor (Gibco/Invitrogen, Grand Island, NY), and 1 mg/mL hyaluronidase (Sigma-Aldrich) in Ca^++^/Mg^++^-free PBS and incubated for 20 min at room temperature (~25°C). After the peritubular cells were removed in the washing step, the seminiferous tubules were re-dissociated in 10 mL of Enzyme Solution B containing 5 mg/mL collagenase, 10 *μ*g/mL DNase I, 1 *μ*g/mL soybean trypsin inhibitor, and 1 mg/mL hyaluronidase in Ca^++^/Mg^++^-free PBS and incubated for 30 min at 37°C. After incubation, the sperm in the dissociated testicular cell samples were removed using a modified two-gradient (35%–70%) Percoll method. The recovered testicular cells were then plated and incubated on uncoated dishes in Germ Cell Culture Medium I, which consisted of DMEM (Gibco/Invitrogen) containing 20% FBS (Gibco/Invitrogen), 10 *μ*mol/L 2-mercaptoethanol (Gibco/Invitrogen), 1% non-essential amino acids (Gibco/Invitrogen), and 10 ng/mL rat GDNF (R&D systems), at 37°C under a humidified atmosphere of 5% CO_2_ in air. Over the following 48 hours, unattached cells were harvested and replated on laminin-coated dishes in Germ Cell Culture Medium II, which consisted of StemPro-34 SFM (Invitrogen) supplemented with 6 mg/mL D(+)glucose, 5 × 10^−5^ M *β*-mercaptoethanol, 1 *μ*M d(L)-lactic acid, 2 mM L-glutamine, 30 *μ*M pyruvic acid, 10^−4^ M ascorbic acid, 60 ng/mL progesterone, 30 ng/mL *β*-estradiol (Sigma-Aldrich), 0.2% BSA (ICN Chemicals), 100 U/mL penicillin, 100 *μ*g/mL streptomycin, 1 × insulin-transferrin-selenium (ITS) supplement, 1 × MEM vitamin solution, 1 × MEM non-essential amino acids, 20 ng/mL mouse EGF, 10 ng/mL human bFGF (Invitrogen), 1% KSR (Invitrogen), 10 ng/mL rat GDNF (R&D systems), and 10^3^ U/mL LIF (Chemicon, Billerica, MA) (modified from [14]). To obtain highly pure SSCs, un-attached cells collected after a 4-h incubation were re-sorted by magnetic activated cell separation (MACS) using an anti-CD9 antibody. The isolated SSCs slowly proliferated and then formed slightly attached clump-like structures (≥10 cells) on the bottom of dishes 2–4 weeks after seeding. During culture, approximately 80% of the medium was changed carefully every other day under a stereomicroscope to avoid the loss of floating clumps. Only clumps were collected. The clumps were dissociated by trypsinization and then re-plated every 2 weeks using the same medium. After every passage, cells clumps were divided into two groups. One group was fixed or sampled for characterization, and the other group was passaged using the method previously described.

### 2.3. Characterization of SSCs from *In Vitro* Culture

To characterize isolated highly pure SSCs and to investigate the relative expression levels of multipotent markers in the SSC clumps, we performed immunocytochemistry using the SSC markers GFR *α*1 (Chemicon International) and CD9 (Chemicon international) and the pluripotent stem cell markers OCT-4 (Santa Cruz), SSEA-4 (Chemicon international), TRA-1-60 (Chemicon international), and TRA-1-81 (Chemicon international). The samples were washed three times in DPBS with 5% FBS and were then fixed in paraformaldehyde (4% v/v in DPBS) for 24 hours. For permeabilization, the cell clumps were incubated in 0.1% Triton X-100 in DPBS for 1 hour. After washing three times with DPBS, the nonspecific binding of antibodies was suppressed by incubating the cells in blocking solution (4% normal goat serum in DPBS) for 30 min at room temperature. After washing three times with PBS, immunocytochemical staining was performed by incubating the fixed samples with primary antibody diluted 1 : 200–1 : 500 with DPBS containing 0.1% Tween-20 and 1% BSA for 60 min at room temperature or overnight at 4°C. Immunoreactive proteins were then detected using CY3- or FITC-conjugated secondary antibodies diluted 1 : 500 with DPBS for 60 min at room temperature. Finally, samples were counterstained with 1 *μ*g/mL 4′,6′-diamidino-2-phenylindole (DAPI; Sigma). Following multiple washes, samples were mounted in Vectashield mounting medium (Vector laboratories, Burlingame, CA). The staining was viewed using an inverted confocal laser scanning microscope (LSM 510; Carl Zeiss, Oberkochen, Germany) with fluorescence at a 400x magnification. Micrographs were stored in LSM (Zeiss LSM Image Browser version 2.30.011; Carl Zeiss Jena GmbH, Jena, Germany).

Alkaline phosphatase activity was assessed by histochemical staining. Cells were fixed in 4% paraformaldehyde at room temperature for 1 min, washed twice with PBS and stained with an alkaline phosphatase substrate solution (10 mL FRV-Alkaline Solution, 10 mL Naphthol AS-BI Alkaline Solution; Alkaline Phosphatase kit, Sigma-Aldrich) for 30 min at room temperature. Alkaline phosphatase activity was detected colorimetrically (red) by light microscopy.

#### 2.3.1. RT-PCR

RT-PCR was performed to assess the expression of multipotent marker genes, specifically, *OCT4, NANOG,* and *Integrin *α*6*, in clumps from SSCs. Total RNA was extracted from 100 colonies using the TRIzol method (Gibco). Amplification was performed in a 20 *μ*L reaction mixture containing 10 mmol/L Tris-HCl (pH 8.3), 2 mmol/L MgCl_2_, 50 mmol/L KCl, 0.25 mmol/L dNTP, 3–5 pmol of each primer, and 1.25 IU Taq polymerase (Gibco). The following genes were amplified using the primers indicated in parentheses: *OCT-4* (F: 5′-GGA AAG GCT TCC CCC TCA GGG AAA GG-3′, R: 5′-AAG AACA TGT GTA AGC TGC GGC CC-3′, 460 bp, GenBank accession number NM002701); *NANOG* (F: 5′-CCC ATC CAG TCA ATC TCA-3′, R: 5′-CCT CCC AAT CCC AAA CAA-3′, 565 bp, GenBank accession number NM024865); *Integrin *α*6* (F: 5′-GGG AGC CTC TTC GGC TTC TC-3′, R: CAC ATG TCA CGA CCT TGC CC-3′, 286 bp, GenBank accession number NM000210) and 18S ribosomal RNA (F: 5′-TAC CTA CCT GGT TGA TCC TG-3′, R: 5′-GGG TTG GTT TTG ATC TGA TA-3′, 255 bp, GenBank accession number K03432). PCR was initiated with a denaturation step at 94°C for 5 min, followed by 35–40 cycles of 30 s at 94°C, 30 s at 55–60°C, and 30 s at 72°C. A final extension step for 10 min at 72°C completed the amplification reaction, after which the products were separated by 1.5% agarose-gel electrophoresis. Negative controls included mock transcription without mRNA and PCR with distilled deionized water.

### 2.4. Flow Cytometry

SSC clumps were dissociated in trypsin-EDTA and resuspended in PBS containing 2% FBS. Then, the cells were incubated with APC-conjugated antibody to SSEA-4 (BD/Pharmingen) for 60 min at 4C. Finally, the cells were placed in the flow cytometer (Becton Dickinson FACS IV San Jose, CA, USA) for analysis. Cells without antibody staining were used as negative controls.

### 2.5. Karyotype Analysis

Chromosome spreads were prepared as described [15]. Briefly, SSCs were treated with 0.06 *μ*g/mL colcemid (Invitrogen) for 2–4 h, trypsinized, incubated in 0.075 M KCl for 10 min, and fixed in Carnoy's fixative. The chromosome number and banding patterns were analyzed with a 300–500 band resolution.

### 2.6. EB Formation from SSCs

After 5 passages, over 200–400 SSC clumps cultured in HEPES-buffered DMEM/F-12 (Gibco) supplemented with 10 *μ*g/mL ITS (Gibco), 10^−4^ mol/L vitamin C (Sigma), 10 *μ*g/mL vitamin E (Sigma), 3.3 × 10^−7^ mol/L retinoic acid (Sigma), 3.3 × 10^−7^ mol/L retinol (Sigma), 1 mmol/L pyruvate (Sigma), 2.5 × 10^−5^ IU recombinant human FSH (Gonal-F; Serono), 10^−7^ mol/L testosterone (Sigma), 1 × antibiotic-antimycotic (ABAM, containing penicillin, streptomycin and amphotericin B; Gibco), and 10% bovine calf serum (Hyclone), for spontaneous *in vitro* differentiation [6]. SSCs clumps were transferred to 1.0 mL of differentiation culture medium in a 24-well dish and were cultured for up to 4 weeks at 37°C in a humidified atmosphere of 5% CO^2^ in air. The medium was replaced on alternate days. After culturing, the EBs were fixed in 10% neutral buffered formalin, embedded in paraffin, stained with hematoxylin and eosin (H&E) and examined immunocytochemically. The endoderm marker *α*-fetoprotein (Chemicon international), the ectoderm marker nestin (Chemicon international) and the mesoderm marker cardiac troponin I (Chemicon international) were used.

### 2.7. Teratoma Formation from Pluriotent SSCs

Pluripotency was determined by harvesting ~2,000 SSC clumps (~2 × 10^5^ cells) and injecting them subcutaneously into the back of 4- to 8-week-old severe combined immunodeficient (SCID) mice (CB 17 strain; Jackson Laboratory, Bar Harbor, ME) using a sterile 26 G needle. After 12 weeks, the resulting tumors were fixed in 10% neutral buffered formalin, embedded in paraffin, cut into 5-*μ*m serial sections, H&E-stained and immunocytochemically examined. The human specific markers, **α**-fetoprotein (Chemicon international), MAP-2 (Chemicon international) and STEM 121 (Stem-121, Stem Cells Inc., Cambridge, UK) were used.

## 3. Results

### 3.1. Morphology and Karyotype of *In Vitro* Cultured SSCs

**Figure 1:**
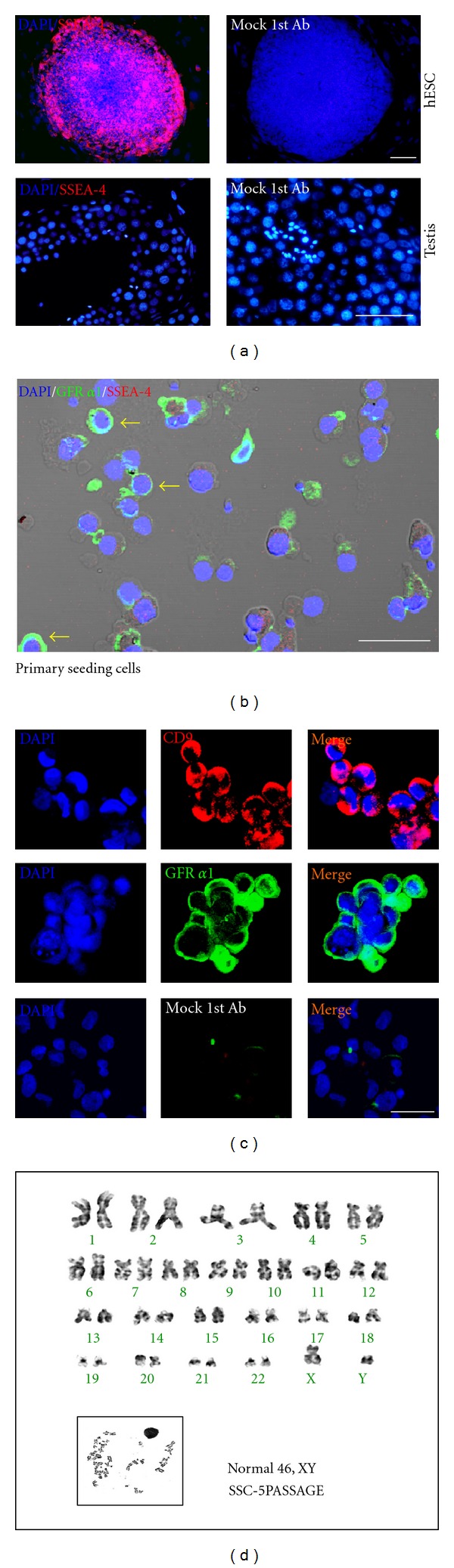
Characterization of spermatogonial stem cells (SSCs). (a) Expression of pluripotent stem cells marker (SSEA-4) in the hESCs and testis. (b) Expression of pluripotent stem cells (SSEA-4) and SSCs marker (GFR *α*1) in the primary seeding cells. The yellow arrows indicate a GFR *α*1-positive signals which were expressed in SSCs. (c) Localization of specific markers for SSCs (CD9, red, and GFR *α*1, green) in the cultured cell clumps (at passage 1). (d) Karyotyping of SSCs performed at passage 5. Note: Mock 1st Ab; cultured cells were stained with secondary antibody only as a negative control. Scale bars = 100 *μ*m.

Significant staining for pluripotent marker (SSEA-4) was detected in hESCs. But testicular tissue did not express this marker (Figure 1(a)). In the primary culture after enzyme treatment, seeding cells exhibited positive signal of GFR **α**1. However, SSEA-4, a pluripotent marker, was not detected in those cells (Figure 1(b)). After the selection procedure, the isolated and cultivated SSCs exhibited high expression levels of SSC marker, as described in our previous report [14]. Significant staining for SSC markers (CD9 and GFR **α**1) was detected at a high level in the SSC clumps (Figure 1(c)). SSCs were well maintained and proliferated in culture, ultimately forming small clumps (>10 cells), and were passaged by trypsin-dissociation and plating on new culture dishes with fresh medium containing GDNF. SSCs attached to the plate after incubation for 2-3 days and then proliferated by re-forming floating clumps. Somatic cells and differentiated cells attached to the dish. The passaging of floating clumps was repeated every two weeks. Dissociated SSCs continued to proliferate for more than 5 passages (>10 weeks) and re-formed floating clumps. Using this method, we successfully isolated SSCs and maintained proliferating SSC cultures from more than 83.3% (15/18) of OA patients.

To determine the chromosome stability of SSC clumps, karyotyping analysis was performed at passage 5. The results demonstrated that the SSC clumps had a normal karyotype (46, XY), and no indications of other cytogenetic abnormalities were detected (Figure 1(d)).

### 3.2. Immunocytochemical Staining

**Figure 2:**
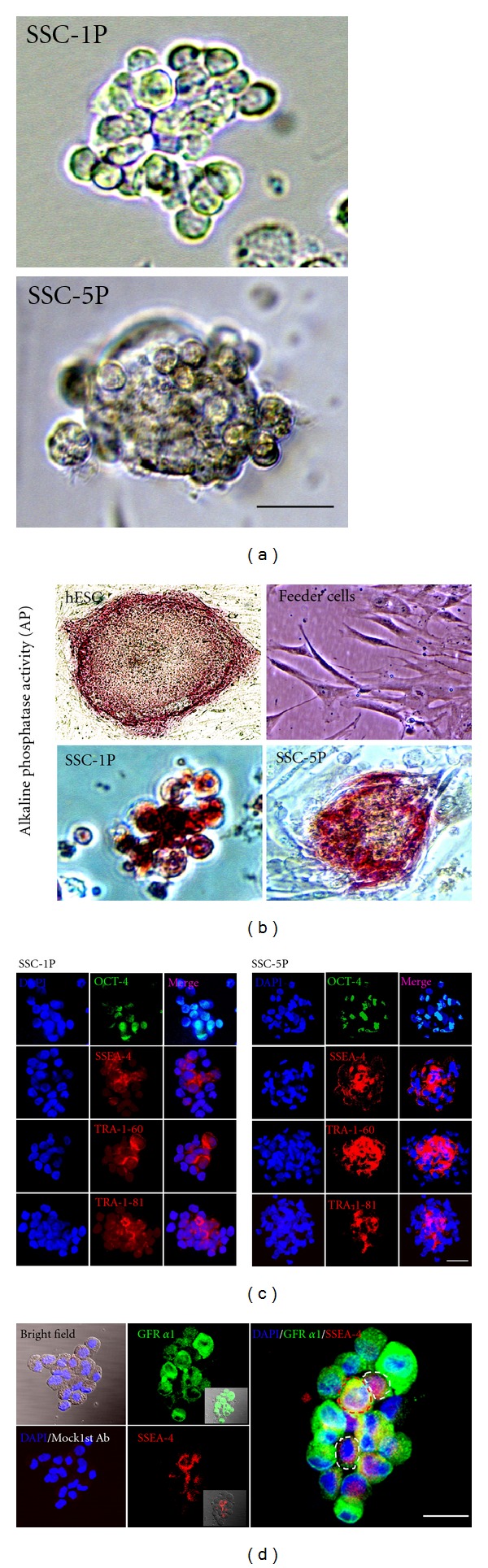
Characterization of pluripotent stem cells within spermatogonial stem cell (SSC) clumps. (a) Morphology of spermatogonial stem cell (SSC) clumps after *in vitro* culture (upper: passage 1, lower: passage 5). (b) Alkaline phosphatase activity in SSC colonies after culture (passage 1 and passage 5). CHA-hES4 cells (human embryonic stem cell line, hESCs) were used as a positive control, and feeder cells were used as a negative control. (c) Immunocytochemical analysis of pluripotent stem cell markers (OCT4, SSEA-4, TRA 1–60 and TRA 1–81) was performed with SSC clumps at passage 1 (left panel) and passage 5 (right panel). (d) Colocalization of specific markers for pluripotent stem cells (SSEA-4, red color) and SSCs (GFR *α*1, green color) in the cultured SSC clumps (at passage 1). The red circle indicates a SSC in which both markers were co-expressed. The yellow circles indicates a mSSC in which pluripotent stem cell-marker were only expressed. Note: Mock 1st Ab; cultured cells stained with secondary antibody only as a negative control. Scale bars = 50 *μ*m.

The morphology of SSC clumps was flattened and loosely associated at first (upper panel of Figure 2(a)) and then changed to tightly associated clumps (lower panel of Figure 2(a)). High levels of AP activity were associated with multicellular clumps *in vitro* (Figure 2(b)).  Figure 2(c) summarizes the expression of pluripotent stem cell markers in the SSC clumps. At passages 1 to 5, immunostaining analysis showed that SSC clumps expressed pluripotent stem cell markers (Oct-4, SSEA-4, TRA-1-60, and TRA-1-81). The expression levels of these markers were slightly increased up to passage 5, but their relative amounts were still low. Additionally, in SSC clumps, some cells expressed the pluripotent stem cell marker SSEA-4, and most expressed a SSC marker, GFR *α*1. Interestingly, a few SSCs co-expressed SSEA-4 and GFR **α**1 (Figure 2(d)).

### 3.3. Flow Cytometric and Gene Expression Analysis of Pluripotent Stem Cell Markers in SSC Clumps

**Figure 3:**
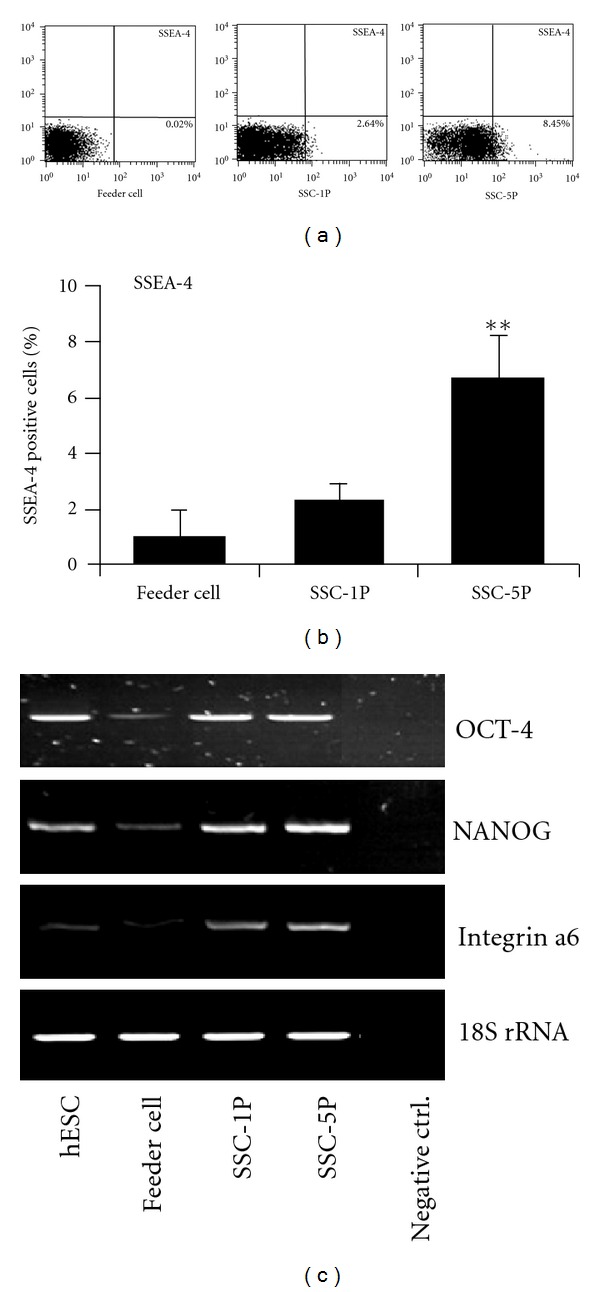
Identification of pluripotent stem cells within spermatogonial stem cell (SSC) clumps using flow cytometry and RT-PCR. (a) Flow cytometric analysis of the cultured SSC clumps using a pluripotent stem cell marker (SSEA-4). (b, c) Immunocytochemical analysis and RT-PCR analysis of pluripotent stem cell markers (*OCT-4*, *NANOG,* and *Integrin *α*6) *in the cultured SSC clumps. 18S ribosomal RNA was used as an experimental control.

The flow cytometric analysis indicated that the number of SSEA-4-positive cells in SSC clumps was greater at passage 5 (8.45%) than at passage 1 (2.64%). These results were similar to the immunostaining results (Figures 3(a) and 3(b)). The gene expression levels of the markers *OCT-4*, *NANOG,* and *Integrin *α*6* were confirmed by RT-PCR. ESC cells and SSC clumps (from passage 1 and 5) strongly expressed the mRNAs for *OCT-4*, *NANOG* and *Integrin *α*6*. In contrast, testicular feeder cells did not express or weakly expressed these genes (Figure 3(c)).

### 3.4. Spontaneously Differentiation *In Vitro*

**Figure 4:**
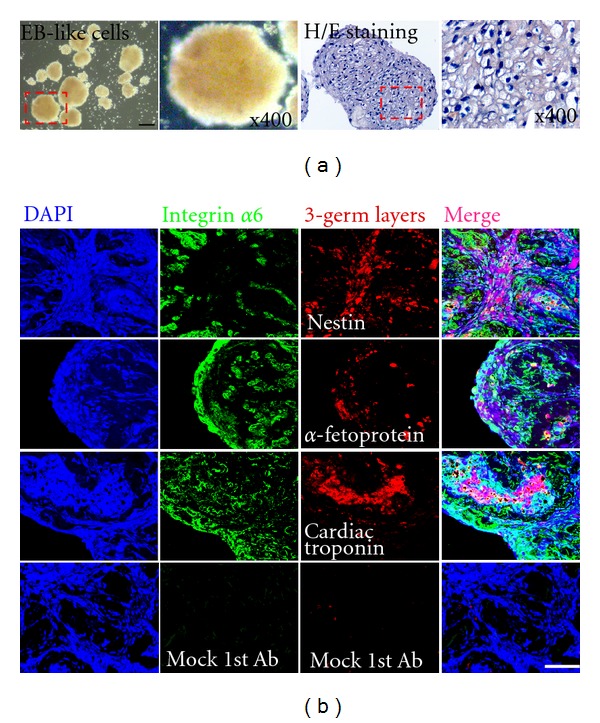
Spontaneous differentiation of spermatogonial stem cell (SSC) clumps *in vitro.* (a) Morphology of differentiated EB-like structured cells after plating onto non-coated dishes, and hematoxylin and eosin staining of EB-like structured cells. (b) Expression of three germ layer-specific markers in EB-like structured cells derived from SSC clumps. Markers of ectodermal progenitor cells (nestin), endodermal lineage cells (*α*-fetoprotein) and mesodermal cells (cardiac protein). Note: Mock 1st Ab; cultured cells stained with secondary antibody only as a negative control. Scale bars = 100 *μ*m.

To investigate the differentiation potential of the SSCs clumps *in vitro*, SSC clumps at passage 5 were spontaneously differentiated using the suspension EB-formation method in the absence of growth factors. After 10–14 days under these culture conditions, the SSC clumps consistently aggregated and formed EB-like structures (Figure 4(a)). Markers of ectodermal progenitor cells (nestin, which is present in neuro-epithelial cells) and endodermal lineage cells (*α*-fetoprotein, expressed in early and late hepatocytes) were detected in the EB-like structures [16, 17]. We also found that marker of mesoderm cells (cardiac protein, widely localized in cardiac muscle cells) was expressed in the EB-like structures and the expression of integrin *α*6, a marker of pluripotent stem cells, was remained after *in vitro* differentiation (Figure 4(b)).

### 3.5. Teratoma Formation Potential of SSC Clumps

**Figure 5:**
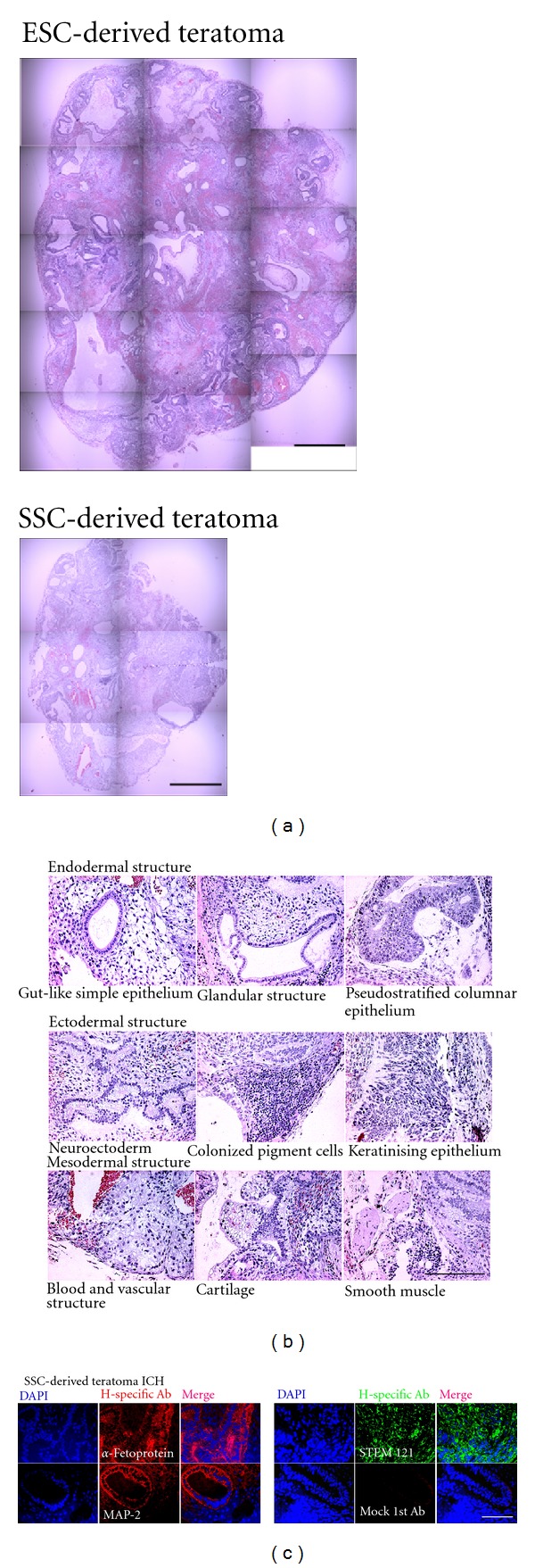
Teratoma formation from transplanted pluripotent stem cells within spermatogonial stem cells (SSCs) (a) Teratoma obtained from hESCs (CHA-hES4) and multipotent stem cells among SSCs. (b) Morphology of three germ layer-like structures obtained from multipotent stem cells within SSCs. (c) Immunohistochemical studies for human specific antibody. *α*-fetoprotein (ectoderm marker reacts with human), MAP-2 (mesoderm marker reacts with human) and STEM121 (cytoplasm marker reacts with human). Note: Mock 1st Ab; cultured cells stained with secondary antibody only as a negative control. Scale bars = 100 *μ*m.

To confirm the *in vivo* differentiation potential, we subcutaneously injected SSC clumps derived from OA patients into the dorsal skin of immune-deficient mice and examined the ability of these cells to form teratomas *in vivo*. Injected SSC clumps gave rise to teratomas in recipients 12 weeks after transplantation. However, wide-scale expansion to large teratomas was not observed, as is typically observed with hESCs (Figure 5(a)). However, the small teratomas contained derivatives of all three embryonic germ layers. Histological analysis revealed that a variety of cell types was present (Figure 5(b)). Using this assay, teratomas were formed in three of ten (30%) injected mouse. In our previous report, 80–90% density SSC-positive cells did not form teratomas or other tumours after transplanatation into SCID mice (data not shown) [14]. Teratomas from the cultured SSC were distinguished from host SCID mouse tissues by human specific antibody (*α*-fetoprotein, MAP-2, and STEM121) (Figure 5(c)).

## 4. Discussion

Although the existence of pluripotent stem cells in the testis has been reported in human models [6–9, 18], the characteristics and origin of these cells have remained uncertain [10–13]. In contrast to mouse mSSCs [3], human pluripotent SSCs were isolated from heterogeneous testicular cells including SSCs and somatic cells that were not well-characterized. In the present study, we confirmed that human pluripotent SSCs with ESC-like characteristics can be derived from highly pure SSCs. These cells underwent culture-induced reprogramming without any genetic manipulation.

In 2003, Kanatsu-Shinohara and his colleagues succeeded in culturing SSCs obtained from postnatal mouse testes. SSCs formed uniquely shaped colonies when cultured in the presence of GDNF [19]. Even after 2 years of culture *in vitro*, the cells had the potential to produce normal offspring [20]. Recent studies also indicate that the self-renewal of these SSCs in rodents (and possibly all mammals) is dependent on bFGF and LIF [21, 22]. Based on these properties, we isolated human SSCs from testes and propagated these cells in a modified *in vitro* culture system based on that for mouse SSCs [14]. In the present study, the generation of pluripotent stem cells was observed in long-term cultures of purified human SSCs with a normal chromosome status, but immunocytochemistry and flow cytometric analysis revealed that the relative amount of these cells was disappointingly low (~10%). It has been suggested that the current culture conditions may not fully support the stable maintenance or propagation of pluripotent SSCs, and other factors provided by ESC culture medium or additional feeder cells may be required [18]. Generally, pluripotent stem cells such as ESCs are plated and cultured on mouse embryonic fibroblast feeder cell layers, and following a brief period of attachment and expansion, the resulting outgrowth is disaggregated and re-plated onto another feeder cell layer [23]. In our culture system, the feeder cells, mouse embryonic fibroblasts, did not affect the proliferation of human pluripotent SSCs (data not shown). Hence, it is important to identify suitable feeder cells for pluripotent SSCs.

The clump shape of SSCs was similar to that of ES cell colonies during long-term culture (Figure 2(a)). Additionally, very small numbers of cells co-expressed GFR *α*1 and SSEA-4, and some cells expressed SSEA-4 (Figure 2(d)). These results are very similar to our previous results in mice indicating that the intermediate state of SSCs has an expression profile more similar to that of pluripotent stem cells than to that of mSSCs, yet the expression of germ cell markers is preserved [24]. These results suggest that the morphological transformation of SSCs and the start of the expression of specific markers indicate that SSCs can be reprogramed during long-term culture under specific conditions.

Kerr et al. have provided some findings regarding the development and differentiation of human germ cells in the fetal testis, including a very small population of PGCs with a molecular signature including OCT4, NANOG, c-Kit, SSEA-1, SSEA-4, and alkaline phosphatase [25]. Our initial characterization showed that a small number of isolated SSCs expressed markers of undifferentiated stem cells such as OCT4, SSEA-4, Tra-1-60 and Tra-1-81 [26, 27]. The population of these cells was slightly larger after passaging (Figures 2(b) and 3) and was then maintained under defined culture conditions. Pluripotent stem cells have the potential to differentiate into nearly all cell types in the human body [28]. *In vitro* and *in vivo*, these cells are able to generate embryoid bodies or teratomas that express marker genes of all three germ layers and develop into different cell types. The capacity of pluripotent stem cells to differentiate into almost all of the cell types of the human body highlights the potentially promising role of these cells in cell replacement therapies for the treatment of human diseases [29]. If the pluripotent stem cells can be derived from existed/reprogrammed human testis cells, these cells will generate embryoid bodies in culture. In our system, pluripotent cells among SSCs were able to generate EB-like structures and exhibited some characteristics of pluripotency, differentiating into cells of the three germ layers (Figure 4). Additionally, these cells formed teratomas after injection into SCID mice (Figure 5).

## 5. Conclusion

This result revealed that pluripotent cells were induced from SSCs during *in vitro *culture and were present within the SSC clumps isolated from human adult testicular tissues. Additionally, no genetic modification was needed for this procedure, which can be compared to the generation of iPSCs from somatic cells [11]. The isolation and long-term proliferation of mSSCs from human testicular tissue may allow the development and use of individual cell-based therapies without ethical and immunological problems. In this study, we demonstrated that adult SSCs are able to develop into pluripotent stem cells in *in vitro* culture, which differentiate into cells of the three germ layers. However, before pluripotent stem cells derived from SSCs can be used in treatments, a highly efficient system to induce and propagate these cells on a large scale is required.
